# Full-Genome Sequencing and Confirmation of the Causative Agent of Erythrocytic Inclusion Body Syndrome in Coho Salmon Identifies a New Type of Piscine Orthoreovirus

**DOI:** 10.1371/journal.pone.0165424

**Published:** 2016-10-27

**Authors:** Tomokazu Takano, Akatsuki Nawata, Takamitsu Sakai, Tomomasa Matsuyama, Takafumi Ito, Jun Kurita, Sachiko Terashima, Motoshige Yasuike, Yoji Nakamura, Atushi Fujiwara, Akira Kumagai, Chihaya Nakayasu

**Affiliations:** 1 Research Center of Fish Diseases, National Research Institute of Aquaculture, Japan Fisheries Research and Education Agency, Minami-Ise, Mie, Japan; 2 Miyagi Prefecture Fisheries Technology Institute, Ishinomaki, Miyagi, Japan; 3 Research Center for Bioinformatics and Biosciences, National Research Institute of Fisheries Science, Japan Fisheries Research and Education Agency, Yokohama, Kanagawa, Japan; GERMANY

## Abstract

Erythrocytic inclusion body syndrome (EIBS) causes mass mortality in farmed salmonid fish, including the coho salmon, *Onchorhynchus kisutchi*, and chinook salmon, *O*. *tshawytscha*. The causative agent of the disease is a virus with an icosahedral virion structure, but this virus has not been characterized at the molecular level. In this study, we sequenced the genome of a virus purified from EIBS-affected coho salmon. The virus has 10 dsRNA genomic segments (L1, L2, L3, M1, M2, M3, S1, S2, S3, and S4), which closely resembles the genomic organization of piscine orthoreovirus (PRV), the causative agent of heart and skeletal inflammation (HSMI) in Atlantic salmon and HSMI-like disease in coho salmon. The genomic segments of the novel virus contain at least 10 open reading frames (ORFs): lambda 1 (λ1), λ2, λ3, mu 1 (μ1), μ2, μNS, sigma 1 (σ1), σ2, σ3, and σNS. An additional ORF encoding a 12.6-kDa protein (homologue of PRV p13) occurs in the same genomic segment as σ3. Phylogenetic analyses based on S1 and λ3 suggest that this novel virus is closely related to PRV, but distinctly different. Therefore, we designated the new virus ‘piscine orthoreovirus 2’ (PRV-2). Reverse transcription–quantitative real-time PCR revealed a significant increase in PRV-2 RNA in fish blood after the artificial infection of EIBS-naïve fish but not in that of fish that had recovered from EIBS. The degree of anemia in each fish increased as the PRV-2 RNA increased during an epizootic season of EIBS on an inland coho salmon farm. These results indicate that PRV-2 is the probable causative agent of EIBS in coho salmon, and that the host acquires immunity to reinfection with this virus. Further research is required to determine the host range of PRV species and the relationship between EIBS and HSMI in salmonid fish.

## Introduction

Mass mortality from erythrocytic inclusion body syndrome (EIBS) occurs frequently and causes severe economic losses on coho salmon (*Onchorhynchus kisutchi*) farms in Japan [1]. The disease was first described in 1982 in juvenile chinook salmon (*O*. *tshawytscha*) reared in a freshwater hatchery in Washington, USA [2]. Variable susceptibility among salmonid fish species, including chum salmon (*O*. *keta*), rainbow trout (*O*. *mykiss*), and masou salmon (*O*. *masou*) [3], was demonstrated by their artificial infection with infected blood homogenates. Infection with the EIBS-like virus has also been reported in farmed Atlantic salmon (*Salmo salar*) in Ireland, Norway, and Scotland [4–6].

Fish suffering EIBS are always characterized by erythrocytic inclusion bodies and anemia [7]. A histopathological examination of EIBS-affected coho salmon also showed necrosis of the muscle fibers in the cardiac ventricle and atrium [8]. Hyperbilirubinemia and the accumulation of bilirubin in the liver [9] may be one of the causes of the jaundice and yellow-colored livers of diseased coho salmon. The causative agent of EIBS is an icosahedral virus, approximately 70–80 nm in diameter, and the virions are often observed within membrane-bound structures that are frequently associated with the cellular membranes [7, 10]. The virions are also occasionally free in the cytoplasm of erythrocytes or combined within large cytoplasmic vacuoles [10]. The preliminary characterization of this virus with electron microscopy revealed similar characteristics to those of a member of the family *Togaviridae* [11], but the isolation of the virus in a fish cell line has been unsuccessful, preventing its further characterization [12].

The causative agent of heart and skeletal muscle inflammation (HSMI) in farmed Atlantic salmon (*Salmo salar* L.), a piscine orthoreovirus (PRV), also forms inclusion bodies in erythrocytes [13, 14]. It is noteworthy that electron microscopic images of the cytoplasmic inclusions of PRV in Atlantic salmon erythrocytes strongly resemble those reported in coho salmon erythrocytes during EIBS [10, 14, 15]. PRV belongs to the family *Reoviridae*, subfamily *Spinareovirinae*, and has been proposed as a first member of a new genus within the family *Reoviridae* [16]. It is a double-stranded nonenveloped RNA virus with 10 genomic segments [17]. Phylogenetic analyses are often performed with the sequences of genomic segment S1 because more complete sequences of S1 from different PRV isolates are available than sequences of other segments [15]. A phylogenetic analysis of PRV isolates from Atlantic salmon in Norway separated the S1 sequences into four main groups (groups I–IV) [18]. Kibenge *et al*. (2013) [16] grouped the Norwegian PRV isolates into a single genotype, genotype I, with subgenotypes Ia and Ib. The Canadian PRV isolates matched sub-genotype Ia and Chilean PRV isolates from Atlantic salmon matched sub-genotype Ib [16]. This classification appears to be very stable because further Norwegian PRV segment S1 sequences [18] supported this grouping [15, 19].

The detection of PRV genomic segments in chinook salmon, coho salmon, and sea trout (*S*. *trutta* L.) [18–20] implies that PRV infection not only occurs in Atlantic salmon but also in other salmonid fish species. HSMI-like diseases caused by PRV-related viral infections have also been reported in rainbow trout in Norway [21]. Recently, HSMI-like disease in the presence of PRV has been reported in Chilean coho salmon [19], which showed lesions in yellow livers, pale gills, splenomegaly, jaundice, hemopericardium, epicarditis, and myocarditis [19]. These pathological changes seem to be consistent with the lesions of EIBS-affected coho salmon in Japan [1, 8, 9]. Based on genomic segment S1 sequences, the Chilean PRV isolates from coho salmon were more genetically diverse (subgenotypes Ia and Ib) than those from Atlantic salmon, and some formed a distinct new phylogenetic cluster, designated genotype II [19]. Notably, the PRV-related virus reported from Norwegian rainbow trout [21] was also classified as genotype II [19].

These findings led us to hypothesize that the EIBS observed in Japanese coho salmon is associated with a PRV or PRV-related infection. However, at present, no molecular data are available on the causative agent of EIBS in coho salmon, and its relationship with HSMI-like disease in coho salmon is unclear. In this study, we determined the genomic sequence and taxonomic position of the causative agent of EIBS in farmed coho salmon in Japan. PCR methods were established to analyze whether this uncharacterized virus correlates with outbreaks of EIBS.

## Results

### Virus purification

After the isolated virus was ultracentrifuged in a sucrose gradient, three bands were visible and fractionated (Figure A in S1 Fig). The symptoms of EIBS were reproduced by inoculating naïve coho salmon with each of three fractions (data not shown). A sodium dodecyl sulfate polyacrylamide gel electrophoresis (SDS-PAGE) analysis of the upper fraction revealed five major proteins (> 25 kDa). A tandem mass spectrometry (MS/MS) analysis showed that four of the five major proteins revealed significant matches to structural proteins of PRV (Figure B in S1 Fig).

### Characterization of the viral genome segments

The novel virus within the upper fraction shared genomic characteristics with members of the family *Reoviridae*. Approximately 2.5 kbp and 4.0 kbp of double-stranded RNA (dsRNA), which were assumed to be medium and large genomic segments, respectively, were detected in the extracted dsRNA (S2 Fig). No dsRNA corresponding in size to small segments (1.0–1.3 kbp) was detected (S2 Fig), but cDNAs corresponding to small, medium, and large segments were successfully amplified with the full-length amplification of cDNAs (FLAC) method (S3 Fig). A sequence analysis of the cDNA amplicons showed that the genome of the novel virus is 23,308 nt in length (GC content, 49%), and consists of 10 dsRNA segments: L1, L2, L3, M1, M2, M3, S1, S2, S3, and S4 (Table 1).

**Table 1:** Summary of PRV-2 genome segments.

Segment*^1^	GenBank accession no.	Size (of PRV)			Encoding protein	Functional properties of PRV	% identity of amino acid sequence to the counterpart proteins in representative members of Spinareovirinae*^2^						
		Segment (bp)	ORF (bp)	Protein (aa)			PRV (genotype Ia)	PRV (genotype Ib)	LMBRV	GCRV104	ARV	MRV	MAHLV
L1	LC145610	3,903 (3,911)	3,837	1,279 (1,282)	λ1	Helicase	92.6	92.7	64.4	31.4	32.9	33.4	30.6
L2	LC145608	3,935 (3,935)	3,870	1,290 (1,290)	λ2	Guanylyltransferase	76.9	76.7	42.8	25.7	26.2	27.1	25.8
L3	LC145609	3,918 (3,916)	3,858	1,286 (1,286)	λ3	RNA-dependent RNA polymerase	88.7	88.7	64.7	38.9	43.4	43.7	44.6
M1	LC145612	2,383 (2,383)	2,280	760 (760)	μ2	Minor inner capsid protein	78.2	78.2	42.2	20.2	23.4	25.2	21.8
M2	LC145613	2,176 (2,179)	2,058	686 (687)	μ1	Outer capsid protein, membrane penetration	84.8	84.7	68.1	24.0	25.7	28.4	27.2
M3	LC145611	2,400 (2,403)	2,256	752 (752)	μNS	Nonstructural protein	59.0	59.7	30.0	20.9	20.2	19.4	19.8
S1	LC145616	1,081 (1,081)	990	330 (330)	σ3	Outer capsid protein, zinc metalloprotein	70.9	69.7	35.0	19.5	18.8	17.1	29.0
			363	121 (124)	p13, σ1s	Cytotoxic, integral membrane protein	65.3	64.5	40.5	NT	NT	NT	NT
S2	LC145614	1,330 (1,329)	1,260	420 (420)	σ2	Inner capsid protein	76.4	77.1	48.1	23.6	23.4	22.9	23.6
S3	LC145615	1,143 (1,143)	1,062	354 (354)	σNS	Nonstructural protein	85.0	84.5	47.7	20.3	22.0	18.2	22.8
S4	LC145617	1,039 (1,040)	945	315 (315)	σ1	Virus attachment protein	66.3	66.3	23.9	19.6	26.1	21.0	NT

NT = not tested. The proteins corresponding to p13 of PRV evolved divergently in other reoviruses. MAHLV lacks an identified cell attachment protein in the smallest segment (S4).

*^1^ Segment IDs were assigned according to the nomenclature used by Palacios *et al*. (2010) [17].

*^2^ Representative reoviruses, including piscine orthoreovirus (PRV genotypes Ia and Ib), grass carp reovirus (GCRV104), *Avian orthoreovirus* (ARV), and *Mammalian orthoreovirus* (MRV), were selected. Novel members of *Spinareovirinae*, including largemouth bass reovirus (LMBRV) and Mahlapitsi virus (MAHLV), were also analyzed. See S1 Table for GenBank accession numbers.

All the genome segments contain conserved nucleotides at the 3´-terminus (UCAUC-3´) and 5´-terminus (5´-GAUAAA/U) that are identical to those in the PRV genome [17, 22]. These segments contain open reading frames (ORFs) encoding lambda 1 (λ1), λ2, λ3, mu 2 (μ2), μ1, μNS, sigma 3 (σ3), σ2, σNS, and σ1, respectively (Table 1). We noted that the segment sizes of L1, L3, M2, M3, and S2 of the novel virus differ slightly from those of PRV (Table 1). Segment S1 is probably polycistronic, because an additional ORF encoding a 12.6-kDa protein (homologue of PRV p13) was identified in this segment. At the amino acid level, the proteins of the novel virus share the highest percentage identities with those of PRV, within a range of 59.0%–92.7% (Table 1). However, no large differences in the percentage identities were detected when the novel virus was compared with PRV genotypes Ia and Ib (Table 1). The amino acid sequence similarities between our virus and largemouth bass reovirus (LMBRV) [23], a newly identified member of the piscine orthoreoviruses, range from 23.9%–68.1% (Table 1). Thus, the novel virus appears to be more closely related to PRV than to LMBRV.

**Fig 1:**
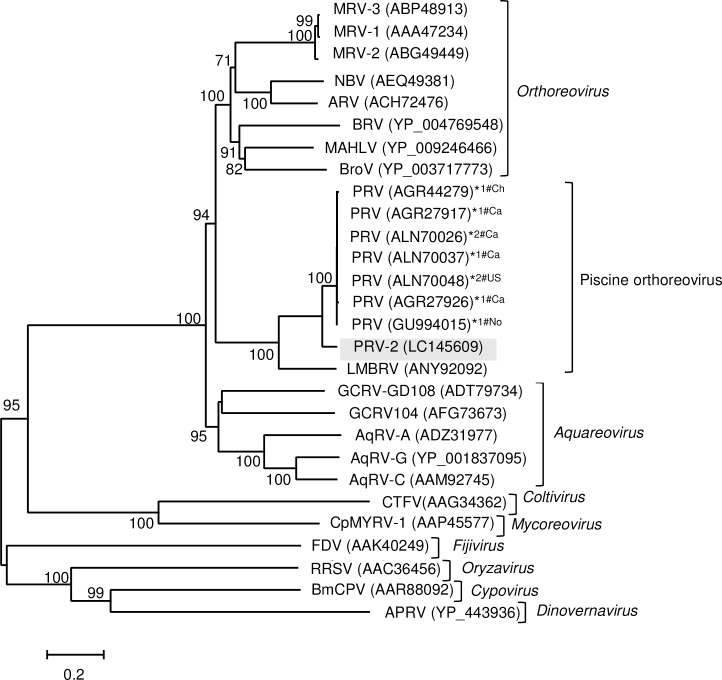
Phylogenetic analysis of the RNA-dependent RNA polymerase (RdRp) of the novel virus, PRV, and members of the subfamily *Spinareovirinae*. Phylogenetic tree was constructed with the amino acid sequences of the putative RdPds of representative viruses from the following genera in the subfamily *Spinareovirinae*: *Aquareovirus*: aquareovirus A (AqRV-A), aquareovirus B (AqRV-B), aquareovirus C (AqRV-C), grass carp reovirus strain 104 (GCRV104), and grass carp reovirus Guangdong 108 strain (GCRV-GD108); *Coltivirus*: *Colorado tick fever virus* (CTFV); *Cypovirus*: *Bombyx mori* cytoplasmic polyhedrosis virus (BmCPV); *Dinovernavirus*: *Aedes pseudoscutellaris* reovirus (APRV); *Fijivirus*, *Fiji disease virus* (FDV); *Mycoreovirus*: *Cryphonectria parasitica* mycoreovirus 1 (CpMYRV-1); *Orthoreovirus*: *Avian orthoreovirus* (ARV), *Baboon orthoreovirus* (BRV), *Broome virus* (BroV), Mahlapitsi virus (MAHLV), *Mammalian orthoreovirus* serotype-1 (MRV-1), *Mammalian orthoreovirus* serotype-2 (MRV-2), *Mammalian orthoreovirus* serotype-3 (MRV-3), and *Nelson Bay orthoreovirus* (NBV); *Oryzavirus*: *Rice ragged stunt virus* (RRSV); Piscine orthoreoviruses: piscine orthoreovirus (PRV), piscine orthoreovirus 2 (PRV-2), and largemouth bass reovirus (LMBRV). The novel virus (PRV-2) is indicated by a shaded box. The accession numbers of the RdRp sequences follow the virus names. RdRp sequences of PRV isolates collected from different hosts (Atlantic salmon [*1] and coho salmon [*2]) and in different places (Canada [#Ca], Chile [#Ch], Norway [#No], or United States [#US]) were analyzed. The tree was constructed with the neighbor-joining method. Numbers on the nodes represent the confidence limits (> 70%) estimated from 1,000 bootstrap replicates.

**Fig 2:**
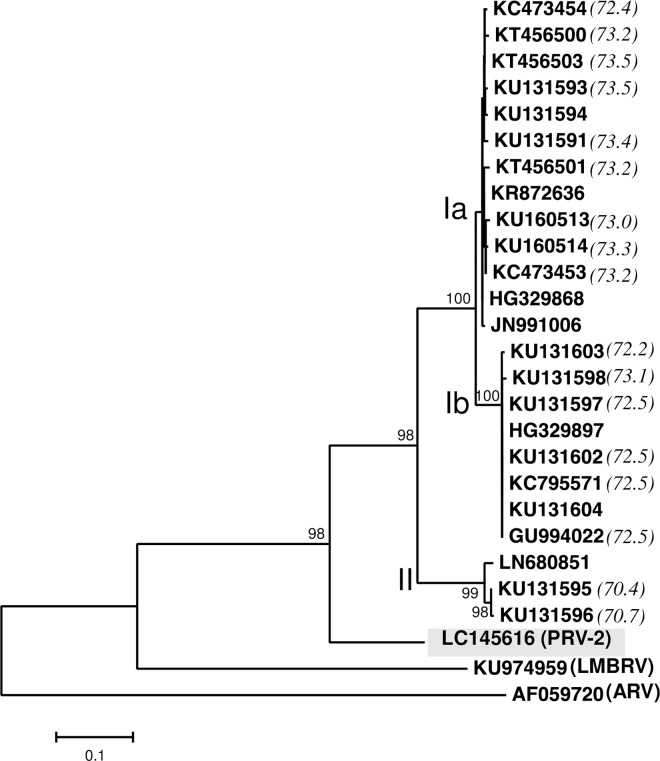
Phylogenetic analysis of segment S1 sequences from the novel virus, PRV genotype I and genotype II isolates, and LMBRV. Phylogenetic tree was constructed with the nucleotide sequences of segment S1 from the novel virus (LC145616), PRV isolates (subgenotype Ia, subgenotype Ib, and genotype II) and LMBRV (KU974959). The novel virus (PRV-2) is indicated by a shaded box. An outgroup (ARV: AF059720) was used to determine the root. The tree was constructed with the neighbor-joining method. Numbers on the nodes represent the confidence limits (> 70%) estimated from 1,000 bootstrap replicates. Percentage identities between the novel virus (LC145616) and the complete nucleotide sequence of PRV segment S1 (1,081 bp) are indicated in parentheses.

A phylogenetic tree constructed from the amino acid sequences of λ3 (RNA-dependent RNA polymerase, RdRp) indicated that the novel virus forms a genetic lineage with the piscine orthoreoviruses (PRV and LMBRV), which is distinct from the clades of *Orthoreovirus* and *Aquareovirus* (Fig 1). It is noteworthy that the RdRp sequence of our virus (LC145609) clustered distantly from other known sequences of PRV (ALN70026 and ALN70048) that were isolated from coho salmon collected on the Canada/USA Pacific coast (Fig 1). Furthermore, the segment S1 sequence of the novel virus clearly differed from the sequences of PRV isolates belonging to subgenotype Ia, subgenotype Ib, and genotype II (Fig 2). The percentage identities between the nucleotide sequences of segment S1 of the novel virus and PRV isolates were 70.4%–73.5% (Fig 2), whereas the sequence identities between the PRV isolates (subgenotype Ia, subgenotype Ib, and genotype II) were ≥ 79.0% [19], suggesting that our virus has a novel segment S1.

These results indicate that the virus purified from EIBS-affected erythrocytes is a member of the piscine orthoreoviruses. The virus is more closely related to PRV isolates than to LMBRV, but is genetically distinct from known PRV isolates. Therefore, we tentatively designated this novel virus, which is presumed to be associated with EIBS in coho salmon in Japan, ‘piscine orthoreovirus 2’ (PRV-2).

### Increased viral RNA load in peripheral blood after artificial PRV-2 infection

**Fig 3:**
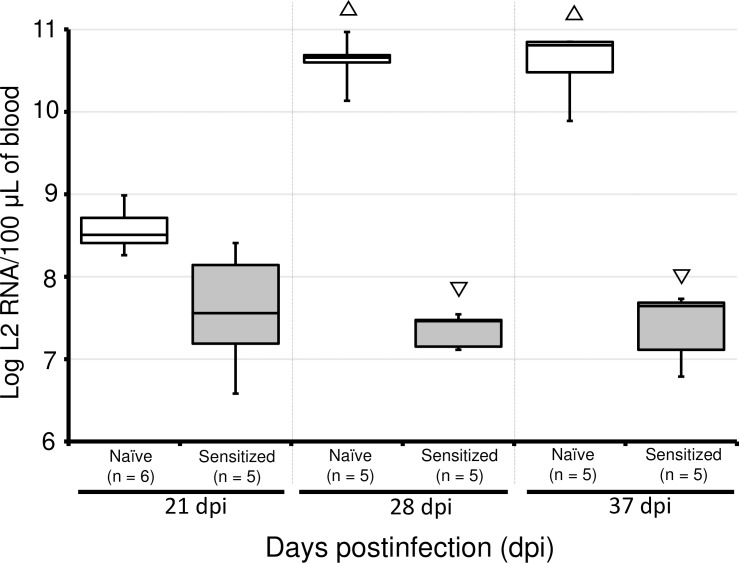
Comparison of time-course changes in the viral RNA loads in EIBS-sensitized and EIBS-naïve coho salmon after artificial infection. Nonparametric analysis of multiple comparisons with Steel’s test was used to determine statistical significance (*p* < 0.05). Viral RNA loads are given as the copy number of L2 RNA per 100 μL of peripheral blood. The copy number of L2 RNA was normalized to the copy number of the external standard RNA (λpolyA^+^ RNA). Upward triangle (△) indicates a significant increase in the viral RNA load, and downward triangle (▽) indicates a significant reduction in the viral RNA load compared with the viral RNA load in the naïve fish at 21 dpi. Box plots show median, 25th, and 75th percentiles, and whiskers represent minimum and maximum values.

The conventional PCR developed in this study detected the targeted region of segment L1 in cDNA samples from EIBS-affected coho salmon (data not shown). Fish negative for PRV-2 infection were identified with conventional PCR and were used for the study. The PRV-2 RNA loads in the peripheral blood (PB) of EIBS-naïve coho salmon and fish that had recovered from EIBS (EIBS-sensitized coho salmon) after artificial infection were examined with reverse transcription–quantitative real-time PCR (RT–qPCR) assays targeting the transcripts and genomic sequence of the L2 segment (L2 RNA). The copy numbers of L2 RNA at 21 days postinfection (dpi) did not differ between the two experimental groups, whereas at 28 and 37 dpi, the copy numbers of L2 RNA were significantly higher in the naïve fish than in the sensitized fish (multiple comparison with Steel’s test, *p* < 0.05) (Fig 3). The highest L2 RNA load in a naïve fish was 9.3 × 10^10^ copies per 100 μL of PB at 28 dpi. The frequencies of cytoplasmic inclusion body (CIB)-positive fish among the naïve fish at 21, 28, and 37 dpi were 16.7%, 100%, and 100%, respectively. In contrast, no significant change in the copy number of L2 RNA was detected in the EIBS-sensitized fish during the experimental period, and no CIB-positive fish was detected in this group. The highest L2 RNA load in a fish in the EIBS-sensitized group was 2.6 × 10^8^ copies per 100 μL of PB at 21 dpi. Notably, no death occurred in either experimental group during the experiment.

### Kinetics of viral RNA load and hematocrit levels on an inland farm

**Fig 4:**
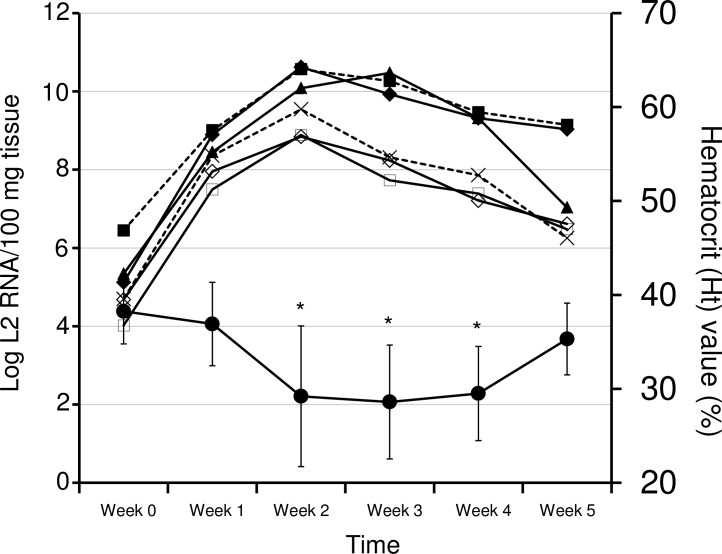
Time-course kinetics of the viral RNA loads in different tissues and the degrees of anemia during an EIBS epizoosis. Mean (n = 5) copy numbers of L2 RNA and hematocrit (Ht) values are shown in the graph. Heart (▲), intestine (◇), kidney (◆), liver (× with broken line), muscle (◻), and spleen (■ with broken line) were tested for viral RNA loads. Viral loads are shown as the copy number of L2 RNA per 100 mg of tissue. Ht values are shown with closed circles (●). Error bars for the Ht values represent standard deviations. ANOVA with the Tukey–Kramer method was used to confirm significant changes in the Ht values (*p* < 0.05). Asterisks indicate significantly reduced Ht values.

**Fig 5:**
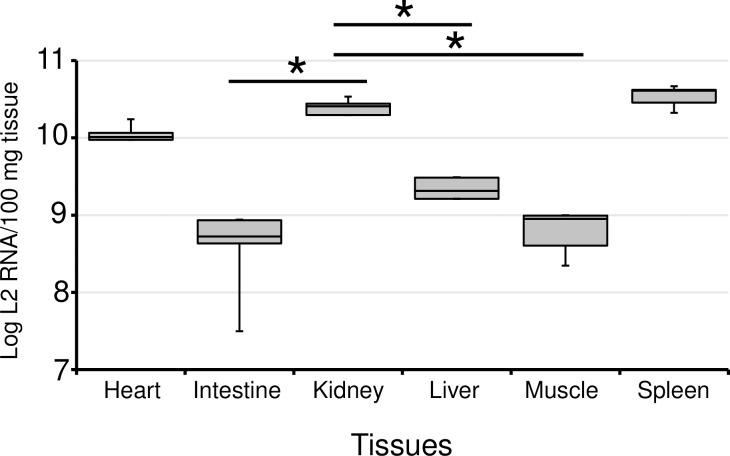
Comparison of the viral RNA loads in different tissues in week 2 of EIBS epizoosis. Viral RNA loads are shown as the copy number of L2 RNA per 100 mg of tissue. Box plots show median, 25th, and 75th percentiles, and whiskers represent minimum and maximum values. ANOVA with the Tukey–Kramer method was used to confirm significant differences in copy numbers. The viral RNA load was significantly higher (*p* < 0.05) in the kidney tissue than in the intestine, liver, or muscle (asterisks).

The time course of changes in the L2 RNA loads in the tissues of fish and their degree of anemia during an epizootic season of EIBS on an inland coho salmon farm were examined with the RT–qPCR assay and hematocrit (Ht) values, respectively. The mean copy numbers of L2 RNA peaked in the intestine, kidney, liver, muscle, and spleen in week 2, whereas the copy numbers peaked in the heart in week 3 (Fig 4). Interestingly, the mean Ht values decreased significantly from week 2 to week 4 (one-way analysis of variance [ANOVA], *p* < 0.05), coinciding with the increased copy numbers of L2 RNA in the tissues (Fig 4). The highest mean copy numbers of L2 RNA in the heart, intestine, kidney, liver, muscle, and spleen during the experiment were 3.0 × 10^10^, 7.0 × 10^8^, 4.2 × 10^10^, 3.5 × 10^9^, 7.7 × 10^8^, and 3.7 × 10^10^ copy per 100 mg of tissue, respectively. Thus, the heart, kidney, and spleen had relatively greater loads of L2 RNA than the other tissues in week 2 (Fig 5). Importantly, the L2 RNA load in the kidney was significantly higher than that in the intestine, liver, or muscle (one-way ANOVA, *p* < 0.05). Notably, the cumulative mortality was estimated to be 23% during this epizootic event, and no death from other diseases was observed.

### Detection of PRVs from EIBS-affected coho salmon

The polymorphic regions of segment S1 in PRV-2, PRV genotype I, and PRV genotype II were successfully identified. Specific PCR primer pairs were designed to amplify the PRV genomes at these regions (Figure A in S4 Fig). A PCR primer set specific for PRV-2 detected segment S1 in cDNA samples from the purified virus, artificially infected coho salmon at 28 dpi, and EIBS-affected coho salmon from the inland farm in week 2 of epizoosis. The PCR primer sets specific for PRV-2 detected neither genotype I nor genotype II in these cDNA samples (Figure B in S4 Fig).

## Discussion

EIBS was first described in hatchery-reared chinook salmon in the 1980s [2], and later in coho salmon [7]. EIBS is characterized by the presence of viral inclusion bodies in the host erythrocytes, and electron microscopy showed the causative agent to have an icosahedral virion, approximately 70–80 nm in diameter [2, 10]. Finstad *et al*. (2014) [14] reported that the particles and inclusions of PRV (the presumptive etiological agent of HSMI) have a striking resemblance to several previously reported viral inclusions described in EIBS. However, it was unclear whether EIBS and HSMI are caused by one virus or several related or unrelated viruses [14]. In this study, we sequenced the genome of a virus purified from the erythrocytes of EIBS-affected coho salmon, and found that it is closely related to PRV. Therefore, we tentatively designated the novel virus ‘PRV-2’.

PRV and the aquareoviruses, which have 10 and 11 dsRNA genome segments, respectively, are known viral pathogens of aquatic organisms, including fish and possibly shellfish [24, 25]. Consistent with the genomic organization characteristic of PRV, the genome of PRV-2 contains 10 dsRNA segments, and 11 proteins are predicted to be encoded by these segments. PRV-2 encodes homologues of all the genes that are found in PRV (λ1, λ2, λ3, μ1, μ2, μNS, σ1, σ2, σ3, σNS, and p13). Previous studies have suggested that the presence of an integral membrane protein, p13, and two essential outer capsid proteins, σ3 and σ1, has important implications for the unique evolution and taxonomic classification of PRV [22, 26, 27]. In PRV, the p13 membrane protein is encoded on the same segment as the σ3 protein, in a bicistronic segment, whereas the σ1 protein is encoded on a monocistronic segment, rather than on a bi- or tricistronic segment [22, 26, 27]. This characteristic genomic organization is fully conserved in PRV-2 and LMBRV. LMBRV is a new member of the piscine orthoreoviruses isolated from a nonsalmonid fish species and is distantly related to PRV [23]. Therefore, these genomic structural properties may be unique to members of the piscine orthoreoviruses.

The piscine orthoreoviruses represent a distinct genus in the family *Reoviridae*, because the clade containing the two PRVs (PRV and PRV-2) and LMBRV on the phylogenetic tree constructed from RdRp sequences was distantly related to members of the genera *Orthoreovirus* and *Aquareovirus*. This is consistent with the findings of previous studies [16, 23]. The RdRp amino acid sequences of the PRV isolates used for the phylogenetic analysis showed high percentage identities (≥ 99.4%), although the sequences were obtained from different countries (Canada [AGR27917, AGR27926, ALN70026, ALN70037], Chile [AGR44279], Norway [GU994015], and the United States [ALN70048]) and different hosts (Atlantic salmon and coho salmon). In contrast, a comparison of the RdRp sequences of PRV-2 and PRV showed much lower identities (88.7%–89.1%). This observation suggests that PRV-2 is genetically distant from the known PRV isolates and represents a distinct species.

The RdRp sequence of ALN70048 (PRV isolate WSKFH12_14) was detected in coho salmon from the Columbia River in Washington State, USA. Recently, erythrocytes have been shown to be the major target cells of PRV replication, which is accompanied by cytoplasmic inclusions [14], but the inclusions observed in EIBS and PRV-associated infections are indistinguishable because the viral morphology in both cases is reovirus-like particles [15]. It is noteworthy that EIBS has also been observed in salmonids (coho salmon and chinook salmon) reared in hatcheries along the Columbia River and its tributaries in the states of Oregon and Washington [7]. These reports raise questions about the etiology of EIBS in coho salmon. Further research is required to determine whether PRV-2, PRV, or both are associated with the EIBS observed in the farmed and wild coho salmon populating the watershed of the Columbia River.

The nucleotide sequence of PRV-2 segment S1 (LC145616) shared lower identity with the S1 sequences of the PRV isolates than was shared among the PRV segments themselves, and PRV-2 was classified as neither genotype I nor genotype II. A phylogenetic tree revealed that PRV segment S1 of Chilean coho salmon with HSMI-like disease (KU131591, KU131593, KU131595, KU131596, KU131598, and KU131604) was clearly distant from that of PRV-2. An RT–PCR analysis of the PRV genotype of segment S1 suggested that PRV-2 was the cause of EIBS among the farmed coho salmon in this study. These observations imply that EIBS in Japanese coho salmon is caused by a novel PRV (RPV-2), which differs from the causative virus of HSMI-like disease in Chilean coho salmon [15]. Comprehensive surveillance is required to clarify whether PRV-2 is an endemic pathogen of Japan or is prevalent worldwide.

It has been reported that coho salmon that recover from EIBS are much less susceptible to reinfection than EIBS-naïve fish [7]. Furthermore, diseased coho salmon transferred from 6°C to 15°C recovered more rapidly than fish that remained at 6°C [7]. Utilizing these phenomena, we prepared EIBS-sensitized coho salmon to examine the time course of changes in the PRV-2 RNA load and the frequency of CIB-positive fish after artificial infection. Protective immunity against PRV-2 appeared to have been induced in the EIBS-sensitized fish because no significant changes in these parameters were observed after infection. However, the frequency of CIB-positive fish among the EIBS-naïve fish increased dramatically from 21 dpi to 28 and 37 dpi. Simultaneously, a significant increase in the viral RNA (L2 RNA) load was observed at 28 and 37 dpi. Previous studies have also reported no inclusion bodies and lower levels of anemia after the injection of recovered fish with an infectious source prepared from EIBS-infected fish [7, 28]. These findings suggest that PRV-2 replication in erythrocytes causes the formation of inclusion bodies and consequent anemia, and that the acquired resistance to PRV-2 that is induced inhibits the onset of EIBS.

The PRV-2 RNA loads and Ht values both became severe in the first half of the experiment on the coho salmon farm. However, the viral RNA loads started to decline thereafter and the Ht values returned to normal. It is hypothesized that the progression of EIBS has five distinct stages [7]. Following an incubation period, when no pathological changes are evident, inclusion bodies appear and increase in number over time. Soon after the inclusion bodies reach their highest numbers, the infected erythrocytes are lysed and Ht values are lowest. Recovery follows and is reflected in an absence of inclusion bodies and a return to normal Ht values [7]. Our observations appear to support this hypothesis and demonstrate the relationship between the prevalence of PRV-2 and the occurrence of EIBS on this coho salmon farm in Japan.

An RT–qPCR analysis of tissues from EIBS-affected fish demonstrated the pattern of PRV-2 distribution. The virus preferentially infects tissues rich in erythrocytes, such as the blood, kidney, and spleen. Higher PRV-2 RNA loads (> 1.0 × 10^10^ L2 RNA/100 μL PB or 100 mg tissue) were found in these tissues in the diseased fish (either artificially or naturally infected with EIBS). Similarly, in a previous study of PRV, the blood, spleen, and kidney had the highest viral RNA loads of the 11 tissues tested in HSMI-affected Atlantic salmon [29]. It is generally understood that erythrocytes are the target cells of both PRV species (causative agents of EIBS and/or HSMI), because they form inclusion bodies in these cells [2, 7, 8, 12, 14, 30, 31]. An analysis of the incidence of immature erythrocytes and inclusion bodies led to the hypothesis that immature erythrocytes are initially infected with the causative agent of EIBS [32, 33]. A flow-cytometric analysis also showed that more than 50% of erythrocytes in HSMI-affected Atlantic salmon were infected with PRV [14]. Another recent study demonstrated the replication of PRV in Atlantic salmon erythrocytes *ex vivo* [31]. These findings explain why higher viral RNA loads of PRV species are present in the blood and hematopoietic tissues. The hearts of the EIBS-affected fish also had relatively high loads of PRV-2 RNA. A previous study of EIBS-affected fish showed necrosis of the muscle fibers in the cardiac ventricle and atrium [8, 19], and an *in situ* hybridization assay revealed the distribution of PRV RNA throughout the myocardia and endocardia of Atlantic salmon with HSMI [17]. Massive epicarditis and the infiltration of lymphocytic cells into the compact and spongy myocardial layer of the ventricle were observed in PRV-affected salmon [14]. Therefore, the heart may also be affected by infections of PRV species.

Mass mortality was observed during a natural epizootic event involving EIBS on this inland coho salmon farm, although it was not associated with the artificial infection performed in this study. It was assumed that other diseases associated with natural EIBS, primary bacterial cold-water diseases, were responsible for the high mortality [7]. However, we observed no outbreak of any other disease, including bacterial cold-water diseases or bacterial kidney disease. Takahashi *et al*. (1994) [33] reported that the erythrocytic inclusions occurred most frequently in groups of fish fed the largest amount of feed, which consequently grew rapidly. In addition, Ht value of EIBS-affected fish was inversely related to fish growth [33]. The growth of the coho salmon fries in our study was more rapid than that reported by Takahashi *et al*. (1994) [33], and their mean body weight increased from 11 g to 40 g in 60 days (data not shown). Therefore, we hypothesize that the rapid growth of the fries and their PRV-2 infection may have synergistically triggered some physiological disruption, such as anoxia attributable to severe anemia, which then resulted in mass mortality.

The present study suggests that PRV-2 is the probable causative agent of EIBS in coho salmon in Japan. We demonstrated the genetic divergence of PRV-2 from known PRV isolates, so it is reasonable to infer that several types of PRV occur on salmonid fish farms worldwide. Importantly, PRV is thought to have been associated with at least some previously reported episodes of EIBS [14]. Further studies are required to determine the host range of the proposed PRV genus [16] and the association between EIBS, HSMI, and HSMI-like diseases. The genomic segments of the orthoreoviruses are known to be susceptible to genetic reassortment and intragenic rearrangement [34, 35]. The exchange of RNA segments between viruses could lead to viral diversity, with increased virulence and an extended host range [36, 37]. Therefore, the reassortment of piscine orthoreoviruses must be clarified because PRV-2 and PRV infect the same hosts, including the coho salmon.

## Materials and Methods

### Ethics statement

This work meets all the relevant standards for the ethics of experimentation and research integrity. The fish handling, husbandry, and sampling methods were approved by the Institutional Animal Care and Use Committee of the National Research Institute of Aquaculture (IACUC-NRIA no. 27003). The owners of the fish farms gave their explicit permission to work on the premises and to sample the diseased fish that were included in the study.

### Viral source and purification

We sampled the fish during an outbreak of EIBS in a marine net pen on a private coho salmon farm in Miyagi Prefecture, Japan, in 2012. Seventy-five (75) moribund coho salmon were anesthetized by bath immersion in 2-phenoxyethanol before sampling, and the kidneys, spleens, and heparinized blood were collected individually. The average bodyweight of the fish was 815 g. The kidney and spleen tissues were used as the source of infection in the challenge test, and the blood was used to purify the virus. A blood smear was prepared from each sample, and the degree of disease progression was identified according to previously reported criteria [32]. Erythrocytes corresponding to EIBS progression stage III-a [32] were collected by centrifugation from 20 mL aliquots of the blood samples. To burst the erythrocytes, each sample was adjusted to four times its original volume with the addition of distilled water. The supernatant of each lysate was transferred to a new tube after centrifugation at 5,000 × g for 10 min at 4°C, and sonicated with a VCX-500 sonicator (Sonics & Materials, Newtown, CT, USA) at 20 W for 2 min. The sonication and centrifugation steps were repeated twice. The virus in the supernatant was precipitated by the addition of 6.5% (w/v) polyethylene glycol 6000 and 2.3% (w/v) NaCl. The mixture was stirred gently for 2 h at 4°C. After centrifugation at 4,000 × g for 10 min at 4°C, the precipitate was suspended in TNE buffer (20 mM Tris [pH 8.3], 150 mM NaCl, 10 mM EDTA). The suspension was then treated with an equal volume of Vertrel XF (DuPont, Wilmington, DE, USA) to remove the lipids and other interfering substances. The top aqueous layer was then layered on top of a 10%–60% linear sucrose density gradient, and centrifuged for 18 h at 110,000 × g (SW40 rotor; Beckman Coulter, Miami Lakes, FL, USA). The three visible bands near the bottom of the tube were fractionated, diluted in fresh TNE buffer, and centrifuged at 150,000 × g for 1 h at 4°C. The pellet was resuspended directly in TNE buffer and stored at −80°C until analysis.

### Protein identification with MS/MS

The protein bands in an SDS-PAGE gel containing the fractionated samples after ultracentrifugation were excised and subjected to microwave-assisted in-gel trypsin digestion [38, 39]. The digests were extracted from the gel pieces with 50% acetonitrile (ACN) containing 0.1% trifluoroacetic acid (TFA), and then with 75% ACN containing 0.1% TFA. Each extraction was performed for 15 min at room temperature. The mixture of these extracts was dried in a centrifugal concentrator and then dissolved in 50% ACN containing 0.1% TFA. The dissolved sample was mixed with an equal volume of the matrix reagent (dihydroxybenzoic acid; Shimadzu, Kyoto, Japan) for matrix-assisted laser desorption/ionization (MALDI)–MS, and then subjected to an MS/MS analysis with MALDI–quadrupole ion trap–time of flight–MS (Axima Resonance; Shimadzu) at the Mass Spectrometry Facility, Faculty of Fisheries/Joint Research Division, Nagasaki University, Japan. The protein was identified based on the MS/MS data using Mascot MS/MS Ion Search (Matrix Science, London, UK).

### Sequencing the genome segments

The upper band obtained after ultracentrifugation was used to determine the nucleotide sequences of the viral genome segments. The sample was incubated for 10 min at 60°C in lysis buffer (10 mM Tris-HCl [pH 8.0], 1 mM EDTA, 1% SDS) containing proteinase K at a final concentration of 0.1 mg/mL, and then mixed with three volume of TRIzol LS Reagent (Invitrogen, Carlsbad, CA, USA). After chloroform was added and the sample centrifuged, the aqueous phase was collected and mixed with an equal volume of isopropyl alcohol. The total RNA was harvested by centrifugation and washed with 75% ethanol. The dsRNA was then separated from any contaminating single-stranded RNA by precipitating it in 2 M lithium chloride (LiCl). The dsRNA precipitate was collected by centrifugation, washed with 75% ethanol, and dissolved in nuclease-free water. The full-length cDNAs of the viral genome segments were obtained with the FLAC method, according to a previous report [40]. The amplified cDNAs were cloned into the pCR2.1 plasmid vector using the TOPO TA Cloning Kit (Invitrogen), and the nucleotide sequences were determined with an ABI Prism 3130xl Genetic Analyzer (Applied Biosystems, Foster City, CA, USA).

### Phylogenetic analysis

The amino acid sequence of RdRp and the nucleotide sequence of the S1 segment of PRV-2 were determined. The sequences of the RdRps and S1 segments of other reoviruses were downloaded from GenBank. The sequences were aligned with ClustalW [41], and a phylogenetic tree was constructed with the neighbor-joining algorithm, with 1,000 resamplings, in MEGA 7.0 [42].

### Artificial infection

Kidney and spleen tissues were collected from EIBS-affected coho salmon (weight of pooled tissues was 11 g) and homogenized in four volumes of phosphate-buffered saline (PBS) on ice. The supernatant was collected after centrifugation at 13,000 × g for 10 min at 4°C. The pelleted tissue debris was homogenized again in PBS, centrifuged, and the supernatant collected. Both supernatants were mixed and adjusted to 110 mL with PBS, and then treated with an equal volume of Vertrel XF. The supernatant was passed through a 0.45 μm filter and stored at −80°C for use as the source of infection. This preparation was diluted 1:10 in PBS, and 0.1 mL was injected intraperitoneally into coho salmon to artificially induce infection. EIBS-sensitized fish were prepared with a previously reported procedure [28], with some modification. The coho salmon injected with the infectious source were maintained at 8°C for 18 days and then at 15°C for a 15 day recovery period. These EIBS-sensitized fish were acclimated to 8°C for 1 day and were then reinjected with the infectious source. Simultaneously, EIBS-naïve coho salmon were also injected with the infectious source and were treated as the positive control for infection. These fish were maintained at 8°C and their peripheral blood was sampled (n = 5–6) at 21, 28, and 37 dpi to determine the copy numbers of viral RNA with RT–qPCR. Blood smears were prepared from each sampled fish, according to a previous report [32], to examine the frequency of fish with CIB in their erythrocytes. The fish used in this experiment were anesthetized by bath immersion in 2-phenoxyethanol before treatment or sampling.

### Fish sampling on an inland farm

The time course of the changes in the viral RNA loads in different tissues, including the heart, intestine, kidney, liver, muscle, and spleen, was analyzed during an epizootic season of EIBS in 2014. Tissue samples were collected between July 3 and August 8 at intervals of 1 week (6–8 days) from a pond on an inland coho salmon farm in Miyagi Prefecture, Japan. The tissues were excised from the anesthetized fish (n = 5) at each time point, and stored in RNAlater (Qiagen, Hilden, Germany) at −80°C until the RNA was extracted. Peripheral blood was also sampled from the caudal vein of each fish into a heparinized capillary tube to determine the Ht values. The fish had an average body weight of 22.3 g when the last sample was collected. The water temperature was 13°C on the first day of sampling and 16°C on the last day of sampling. The cumulative percentage mortality in the pond during the season was estimated to be 23%, and except for EIBS, no infectious diseases or other mortality-causing agents were detected in the pond.

### RNA extraction from tissues

Total RNA was extracted from 100 mg of tissue (100 μL of peripheral blood) with TRIzol Reagent (Invitrogen). An external control, λpolyA^+^ RNA (1.8 × 10^7^ copies) (Takara Bio, Shiga, Japan), was added to the TRIzol Reagent before the RNA was extracted from each tissue sample. The sample was homogenized with a Multi-beads Shocker (Yasui Kikai, Osaka, Japan) and the total RNA was extracted according to the TRIzol protocol. Each sample of extracted total RNA was dissolved in 100 μL of nuclease-free water, and an aliquot (2 μL) was reverse transcribed.

### Reverse transcription

Reverse transcription was performed with ReverTra Ace qPCR RT Master Mix (Toyobo, Osaka, Japan) in a 10 μL reaction volume. The template RNA was denatured at 95°C for 2 min before reverse transcription at 37°C for 15 min. The reaction was stopped at 98°C for 5 min. The samples containing the first-strand cDNA were stored at −30°C until analysis.

### Primer design

Two PCR primer sets were designed from the nucleotide sequences of the PRV-2 genome segments. For conventional RT–PCR, the primers lambda1-F (5´-GGTGAAGTTCATTCTTGCCAATC-3´) and lambda1-R (5´-ATATCCCGAAGCCTGACAGTCA-3´) were designed based on the nucleotide sequence of the helicase gene (segment L1), and amplified a 300-bp fragment. For RT–qPCR, the primers lambda2-F (5´-CGCTCCTCCAGCAACGAT-3´) and lambda2-R (5´-GGTGGATTGAGGCAGAGTTTG-3´) were designed to amplify a 55-bp fragment of the guanylyltransferase gene (segment L2). A commercial real-time primer for λpolyA (Takara Bio) was used to quantify the cDNA of the external control. The DNA fragments amplified with the lambda1-F/R or λpolyA primers were inserted into the pCR2.1 plasmid vector to generate the standards for quantification.

### Conventional PCR

The PCR primers lambda1-F and lambda1-R were used for a brief diagnostic test of PRV-2 infection with conventional PCR. The PCR was performed with TaKaRa Ex Taq DNA Polymerase, Hot Start Version (Takara Bio). An aliquot (1 μL) of the first-strand cDNA and the PCR primers at final concentrations of 0.4 μM were added to 20 μL of PCR mix. The thermal profile consisted of initial denaturation at 95°C for 3 min, followed by 35 cycles of denaturation of 95°C for 30 s, annealing at 58°C for 30 s, and elongation at 72°C for 30 s, with a final elongation step at 72°C for 3 min. The resulting PCR product was visualized on a 1.5% agarose gel.

### Quantitative real-time PCR

qPCR was performed with the Stratagene Mx3000p qPCR System and the companion software MxPro (Stratagene, CA, USA). An aliquot (2 μL) of the first-strand cDNA and 10 μL of 2 × GeneAce SYBR qPCR Mix α Low ROX (Nippon Gene, Tokyo, Japan) were adjusted to a total volume of 20 μL for qPCR. The gene-specific primers (lambda2-F/R or λpolyA primer) were used at final concentrations of 0.4 μM, and the qPCR was performed under the following cycling conditions: 10 min activation of the Hot-Start Gene *Taq* NT at 95°C, followed by 40 amplification cycles of 95°C for 30 s and 60°C for 1 min. Standard curves were constructed using plasmid vectors containing the target fragments. The copy numbers of these plasmid vector standards were determined based on their molecular weights. The relative viral RNA load was normalized to the copy number of λpolyA^+^ RNA.

### Detection of other PRV types

PCR primer pairs were designed to detect PRV-2 segment S1, PRV segment S1 genotype I (including both subgenotype Ia and subgenotype Ib), or PRV segment S1 genotype II (see Figure A in S4 Fig). The primer pairs PRV-2_1F-1R (forward: 5´-CGACGCCAACACCGGGGGCAGC-3´; reverse: 5´- CCAAAGGCAGGACGCAGGATG-3´) and PRV-2_2F-2R (forward: 5´- CGCCCGACTTCTCTTCTGACCTTGG-3´; reverse: 5´- TTCACAGTACGATCCTCCATCATGTCC-3´) specifically detected genomic segment S1 of PRV-2. Primer pairs PRV-GtI_1F-1R (forward: 5´-AGAAGACAACAGTCGCGGTTCA-3´; reverse: 5´-CCATACGCAGGACGCAGAATG-3´) and PRV-GtI_2F-2R (forward: 5´- TCCCTGACTATTCAACGGAGATGAC-3´; reverse: 5´- CGAAAAGTCATCTCCTCCATTCCAGAG-3´) detected PRV segment S1 genotype I. Primer pairs PRV-GtII_1F-1R (forward: 5´-TGAAGACAACAGTCGTGGTTCC-3´; reverse: 5´- CCATATGCAGGGCGTAGGACA-3´) and PRV-GtII_2F-2R (forward: 5´- TTCCTGATTATTCAACTGAAATGCC-3´; reverse: 5´- CGGAAAGTCATCTCCTCCATGCCACTC-3´) detected PRV segment S1 genotype II. The cDNA samples synthesized from the RNA samples from the purified virus, the blood of naïve coho salmon, the blood of the artificially infected coho salmon at 28 dpi, and the kidneys of coho salmon from the inland farm during an epizootic season of EIBS (at week 2) were analyzed. The PCR mixture was prepared as described for conventional PCR. The thermal profile consisted of initial denaturation at 95°C for 3 min, followed by 35 cycles of denaturation of 95°C for 30 s, annealing at 60°C for 30 s, and elongation at 72°C for 60 s, with a final elongation step at 72°C for 3 min. The resulting PCR product was visualized on a 1.5% agarose gel.

### Statistical analysis

The statistical analyses were performed with the software package Statcel3 (OMS, Tokyo, Japan). One-way ANOVA was performed with the Tukey–Kramer method or multiple comparisons were made with Steel’s test. A value of *p* < 0.05 was considered statistically significant.

## Supporting Information

S1 Fig Bands in a sucrose gradient and SDS-PAGE analysis of the corresponding fractions. A) Supernatant of erythrocyte lysate prepared from EIBS-affected coho salmon was layered onto a linear sucrose density gradient, and subjected to ultracentrifugation. Three bands (U, upper band; M, middle band; and L, lower) were fractionated separately. B) Protein composition of each fraction was analyzed with SDS-PAGE. Tandem mass spectrometry (MS/MS) analysis of the major proteins (electrophoretic bands A, B, C, and D) that were detected in the fraction from the upper band (U) revealed significant matches to structural proteins of PRV. Structural proteins λ1 and λ2 were detected in band A. Structural proteins μ1, σ2, and σ3 were detected in bands B, C, and D, respectively.
(TIF)

S2 Fig Agarose gel electrophoresis of extracted RNAs from the upper band in the sucrose gradient. RNAs were separated on a 1.5% agarose gel in 1 × TBE buffer. Faint bands of approximately 2.5 kbp and 4.0 kbp, corresponding to the medium and large segments of reoviruses, respectively, were detected (indicated with asterisks).
(TIF)

S3 Fig Acrylamide gel electrophoresis analysis of cDNAs generated with the FLAC method. cDNAs were generated from the RNA extracted from the upper band in the sucrose gradient. The cDNAs were separated on a 12.5% acrylamide gel in Tris–glycine buffer. cDNAs corresponding to the sizes of large segments (L), medium segments (M), and small segments (S) were detected.
(TIF)

S4 Fig Detection of segments S1 of PRV-2, PRV genotype I, and PRV genotype II from cDNA samples prepared from the purified virus and EIBS-affected coho salmon. A) Nucleotide sequence alignment of the genomic segment S1 of PRV-2, consensus sequence of segment S1 of PRV genotype I, and consensus sequence of segment S1 of PRV genotype II is shown. Consensus sequence of segment S1 of PRV genotype I, including subgenotype Ia and subgenotype Ib, was generated from sequences determined in a previous study (GenBank accession nos GU994022, HG329848, HG329868, HG329897, JN991006, KC473453, KC473454, KC795571, KR872636, KT456500, KT456501, KT456503, KT456505, KU131591, KU131593, KU131594, KU131597, KU131598, KU131602, KU131603, KU131604, KU160513, and KU160514) [19]. Consensus sequence of segment S1 of PRV genotype II was generated from GenBank accession nos KU131595, KU131596, and LN680851. Regions rich in polymorphisms (1F, 2F, 1R, and 2R) were selected and specific PCR primers designed to them. Identical nucleotides in the aligned sequences are indicated with solid boxes. Arrows indicate the directions of primers. PCR primer pairs targeting 1F and 1R amplified 312-bp fragments. PCR primer pairs targeting 2F and 2R amplified 333-bp fragments. B) Detection of genomic segment S1 in cDNA samples of (i) purified virus, (ii) blood of naïve coho salmon (n = 5), (iii) blood of artificially infected coho salmon at 28 dpi (n = 5), and (iv) kidney of EIBS-affected coho salmon from the inland farm at week 2 (n = 5). Except in the naïve fish, PCR amplicons were detected with the specific primer pairs for PRV-2, whereas no bands were detected with the specific primer pairs for PRV genotype I (Gt I) or Gt II. Molecular size marker (M) indicates 0.5 kbp (upper band) and 0.1 kbp (lower band).
(TIF)

S1 Table List of accession numbers of proteins from representative viruses in the subfamily *Spinareovirinae* used in this study. (DOCX)

## References

[1] Takahashi K, Okamoto N, Kumagai A, Maita M, Ikeda Y, Rohovec JS. Epizootics of erythrocytic inclusion body syndrome in coho salmon cultured in seawater in Japan. J Aquat Anim Health. 1992; 4: 174–181. 10.1577/1548-8667(1992)004<0174:EOEIBS>2.3.CO;2

[2] Leek SL. Viral erythrocytic inclusion body syndrome (EIBS) occurring in juvenile spring chinook salmon (*Oncorhynchus tshawytscha*) reared in freshwater. Can J Fish Aquat Sci. 1987; 44: 685–688. 10.1139/f87-083

[3] Okamoto N, Takahashi K, Maita M, Rohovec JS, Ikeda Y. Erythrocytic inclusion body syndrome: Susceptibility of selected sizes of coho salmon and of several other species of salmonid fish. Fish Pathol. 1992; 27: 153–156. 10.3147/jsfp.27.153

[4] Lunder T, Thorud K, Poppe TT, Holt RA, Rohovec JS. Particles similar to the virus of erythrocytic inclusion body syndrome, EIBS, detected in Atlantic salmon (*Salmo salar*) in Norway. Bull Eur Assoc Fish Pathol. 1990; 10: 21–23.

[5] Rodger HD, Drinan EM, Murphy TM, Lunder T. Observations on erythrocytic inclusion body syndrome in Ireland. Bull Eur Assoc Fish Pathol. 1991; 11: 108–111.

[6] Rodger HD, Richards RH. Observational study of erythrocytic inclusion bodies in farmed Atlantic salmon, *Salmo salar* L., in the British Isles. J fish dis. 1998; 21: 101–112. 10.1046/j.1365-2761.1998.00083.x 29739151

[7] Piacentini SC, Rohovec JS, Fryer JL. Epizootiology of erythrocytic inclusion body syndrome. J Aquat Anim Health. 1989; 1: 173–179. 10.1577/1548-8667(1989)001<0173:EOEIBS>2.3.CO;2

[8] Hayakawa Y, Harada T, Yamamoto M, Hatai K, Kubota SS, Bunya T, et al Histopathological studies on viral anemia in sea-cultured coho salmon (*Oncorhynchus kisutch*). Fish Pathol. 1989; 24: 203–210. .

[9] Sakai T, Murata H, Yamauchi K, Takahashi K, Okamoto N, Kihira K, et al Hyperbilirubinemia of coho Salmon *Oncorhynchus kisutch* infected with erythrocytic inclusion body syndrome (EIBS) Virus. Fish Sci. 1994; 60: 519–521. 10.2331/fishsci.60.519

[10] Michak P, Smith CE, Hopper K. Erythrocytic inclusion body syndrome: a light and electron microscopic study of infected erythrocytes of chinook *Oncorhynchus tshawystscha* and coho *O*. *kisutch* salmon. Dis aquat org. 1992; 12: 229–233.

[11] Arakawa CK, Hursh DA, Lannan CN, Rohovec JS, Winton JR. Preliminary characterization of a virus causing infectious anemia among stocks of salmonid fish in the western United States In: Ahne W, Kurstak E, editors. Viruses of lower vertebrates. Berlin: Springer-Verlag; 1989 p. 442–450.

[12] Meyers TR. First report of erythrocytic inclusion body syndrome (EIBS) in chinook salmon *Oncorhynchus tshawytscha* in Alaska, USA. Dis Aquat Org. 2007; 76: 169–172. 10.3354/dao076169 17760390

[13] Watanabe K, Karlsen M, Devold M, Isdal E, Litlabo A, Nylund A. Virus-like particles associated with heart and skeletal muscle inflammation (HSMI). Dis Aquat Org. 2006; 70: 183–192. 10.3354/dao070183 16903229

[14] Finstad OW, Dahle MK, Lindholm TH, Nyman IB, Lovoll M, Wallace C, et al Piscine orthoreovirus (PRV) infects Atlantic salmon erythrocytes. Vet Res. 2014; 45: 35 10.1186/1297-9716-45-35 . 24694042 PMC4234517

[15] Kibenge FSB, Godoy MG. Reoviruses of aquatic organisms In: Kibenge FSB, Godoy MG, editors. Aquaculture virology. San Diego: Elsevier/Academic Press; 2016 p. 205–236.

[16] Kibenge MJT, Iwamoto T, Wang Y, Morton A, Godoy MG, Kibenge FSB. Whole-genome analysis of piscine reovirus (PRV) shows PRV represents a new genus in family Reoviridae and its genome segment S1 sequences group it into two separate sub-genotypes. Virol J. 2013; 10: 230 10.1186/1743-422x-10-230 . 23844948 PMC3711887

[17] Palacios G, Lovoll M, Tengs T, Hornig M, Hutchison S, Hui J, et al Heart and skeletal muscle inflammation of farmed salmon is associated with infection with a novel reovirus. PLoS ONE. 2010; 5: e11487 10.1371/journal.pone.0011487 . 20634888 PMC2901333

[18] Garseth AH, Ekrem T, Biering E. Phylogenetic Evidence of long distance dispersal and transmission of piscine reovirus (PRV) between farmed and wild Atlantic salmon. PLoS ONE. 2013; 8: e82202 10.1371/journal.pone.0082202 . 24349221 PMC3859594

[19] Godoy MG, Kibenge MJT, Wang YW, Suarez R, Leiva C, Vallejos F, et al First description of clinical presentation of piscine orthoreovirus (PRV) infections in salmonid aquaculture in Chile and identification of a second genotype (Genotype II) of PRV. Virol J. 2016; 13: 98 10.1186/s12985-016-0554-y . 27296722 PMC4906990

[20] Siah A, Morrison DB, Fringuelli E, Savage P, Richmond Z, Johns R, et al Piscine reovirus: genomic and molecular phylogenetic analysis from farmed and wild salmonids collected on the Canada/US Pacific coast. PLoS ONE. 2015; 10: e0141475 10.1371/journal.pone.0141475 . 26536673 PMC4633109

[21] Olsen AB, Hjortaas M, Tengs T, Hellberg H, Johansen R. First description of a new disease in rainbow trout (*Oncorhynchus mykiss* (Walbaum)) similar to heart and skeletal muscle inflammation (HSMI) and detection of a gene sequence related to piscine orthoreovirus (PRV). PLoS ONE. 2015; 10: e0131638 10.1371/journal.pone.0131638 . 26176955 PMC4503464

[22] Markussen T, Dahle MK, Tengs T, Lovoll M, Finstad OW, Wiik-Nielsen CR, et al Sequence analysis of the genome of piscine orthoreovirus (PRV) associated with heart and skeletal muscle inflammation (HSMI) in Atlantic Salmon (*Salmo salar*). PLoS ONE. 2013; 8: e70075 10.1371/journal.pone.0070075 . 23922911 PMC3726481

[23] Sibley SD, Finley MA, Baker BB, Puzach C, Armien AG, Giehtbrock D, et al Novel reovirus associated with epidemic mortality in wild Largemouth Bass (*Micropterus salmoides*). J Gen Virol. 2016 Epub 2016 Aug 5. 10.1099/jgv.0.000568 . 27488948

[24] Attoui H, Fang Q, Jaafar FM, Cantaloube JF, Biagini P, de Micco P, et al Common evolutionary origin of aquareoviruses and orthoreoviruses revealed by genome characterization of Golden shiner reovirus, Grass carp reovirus, Striped bass reovirus and golden ide reovirus (genus *Aquareovirus*, family *Reoviridae*). J Gen Virol. 2002; 83: 1941–1951. 10.1099/0022-1317-83-8-1941 . 12124458

[25] Attoui H, Hertens PPC, Becnel J, Belaganahalli S, Bergoin M, Brussaard CP, et al Reoviridae In: King AMQ, Adams MJ, Carstens EB, Lefkowitz EJ, editors. Virus taxonomy: Ninth report of the international committee on taxonomy of viruses. San Diego: Elsevier/Academic Press; 2012 p.541–637.

[26] Nibert ML, Duncan R. Bioinformatics of recent aqua- and orthoreovirus isolates from fish: evolutionary gain or loss of FAST and fiber proteins and taxonomic implications. PLoS ONE. 2013; 8: e68607 10.1371/journal.pone.0068607 . 23861926 PMC3701659

[27] Key T, Read J, Nibert ML, Duncan R. Piscine reovirus encodes a cytotoxic, non-fusogenic, integral membrane protein and previously unrecognized virion outer-capsid proteins. J Gen Virol. 2013; 94: 1039–1050. 10.1099/vir.0.048637-0 . 23343626

[28] Okamoto N, Takahashi K, Kumagai A, Maita M, Rohovec JS, Ikeda Y. Erythrocytic inclusion body syndrome: resistance to reinfection. Fish Pathol. 1992; 27: 213–216. 10.3147/jsfp.27.213

[29] Garver KA, Johnson SC, Polinski MP, Bradshaw JC, Marty GD, Snyman HN, et al Piscine orthoreovirus from western north America is transmissible to Atlantic salmon and sockeye salmon but fails to cause heart and skeletal muscle inflammation. PLoS ONE. 2016; 11: e0146229 10.1371/journal.pone.0146229 . 26730591 PMC4701501

[30] Hedrick RP, McDowell T, Groff JM. Virus-like particles in erythrocytes of coho salmon (*Oncorhynchus kisutch*). Aquaculture. 1990; 89: 377–381. 10.1016/0044-8486(90)90140-i

[31] Wessel O, Olsen CM, Rimstad E, Dahle MK. Piscine orthoreovirus (PRV) replicates in Atlantic salmon (*Salmo salar* L.) erythrocytes ex vivo. Vet Res. 2015; 46: 26 10.1186/s13567-015-0154-7 . 25888832 PMC4350956

[32] Takahashi K, Okamoto N, Maita M, Rohovec JS, Ikeda Y. Progression of erythrocytic inclusion boby syndrome in artificially infected coho salmon. Fish Pathol. 1992; 27: 89–95. 10.3147/jsfp.27.89

[33] Takahashi K, Okamoto N, Kumagai A, Maita M, Rohovec JS, Ikeda Y. Relationship between fish growth rate and progression of artificially induced erythrocytic inclusion body syndrome in coho salmon, *Oncorhynchus kisutch* (Walbaum). J Fish Dis. 1994; 17: 77–83. 10.1111/j.1365-2761.1994.tb00347.x

[34] Dermody TS, Parker JSL, Sherry B. Orthoreovirus In: Knipe DM, Howley PM, editors. Fields virology, 6th ed. Philadelphia:Lippincott Williams & Wilkins; 2013 p.1304–1346.

[35] Duncan R. Extensive sequence divergence and phylogenetic relationships between the fusogenic and nonfusogenic orthoreoviruses: a species proposal. Virology. 1999; 260: 316–328. 10.1006/viro.1999.9832 . 10417266

[36] Chapell JD, Goral MI, Rodgers SE, dePamphilis CW, Dermody TS. Sequence diversity within the reovirus S2 gene: reovirus genes reassort in nature, and their termini are predicted to form a panhandle motif. J Virol. 1994; 68: 750–756. . 8289378 10.1128/jvi.68.2.750-756.1994 PMC236511

[37] Ouattara LA, Barin F, Barthez MA, Bonnaud B, Roingeard P, Goudeau A, et al Novel human reovirus isolated from children with acute necrotizing encephalopathy. Emerg Infect Dis. 2011; 17: 1436–1444. 10.3201/eid1708.101528 . 21801621 PMC3381585

[38] Juan HF, Chang SC, Huang HC, Chen ST. A new application of microwave technology to proteomics. Proteomics. 2005; 5: 840–842. 10.1002/pmic.200401056 . 15693069

[39] Sun W, Gao S, Wang L, Chen Y, Wu S, Wang X, et al Microwave-assisted protein preparation and enzymatic digestion in proteomics. Mol Cell Proteomics. 2006; 5: 769–776. 10.1074/mcp.T500022-MCP200 . 16339992

[40] Maan S, Rao SJ, Maan NS, Anthony SJ, Attoui H, Samuel AR, et al Rapid cDNA synthesis and sequencing techniques for the genetic study of bluetongue and other dsRNA viruses. J Virol Methods. 2007; 143: 132–139. 10.1016/j.jviromet.2007.02.016 . 17433453

[41] Thompson JD, Higgins DG, Gibson TJ. CLUSTAL W: improving the sensitivity of progressive multiple sequence alignment through sequence weighting, position-specific gap penalties and weight matrix choice. Nucleic Acids Res. 1994; 22: 4673–4680. 10.1093/nar/22.22.4673 . 7984417 PMC308517

[42] Kumar S, Stecher G, Tamura K. MEGA7: molecular evolutionary genetics analysis version 7.0 for bigger datasets. Mol Biol Evol. 2016; 33: 1870–1874. 10.1093/molbev/msw054 . 27004904 PMC8210823

