# Supplementary figures and images for: Full-Genome Sequencing and Confirmation of the Causative Agent of Erythrocytic Inclusion Body Syndrome in Coho Salmon Identifies a New Type of Piscine Orthoreovirus
Source: PLoS One. 2016 Oct 27;11(10):e0165424. doi: 10.1371/journal.pone.0165424 (PMC5082797; doi:10.1371/journal.pone.0165424)

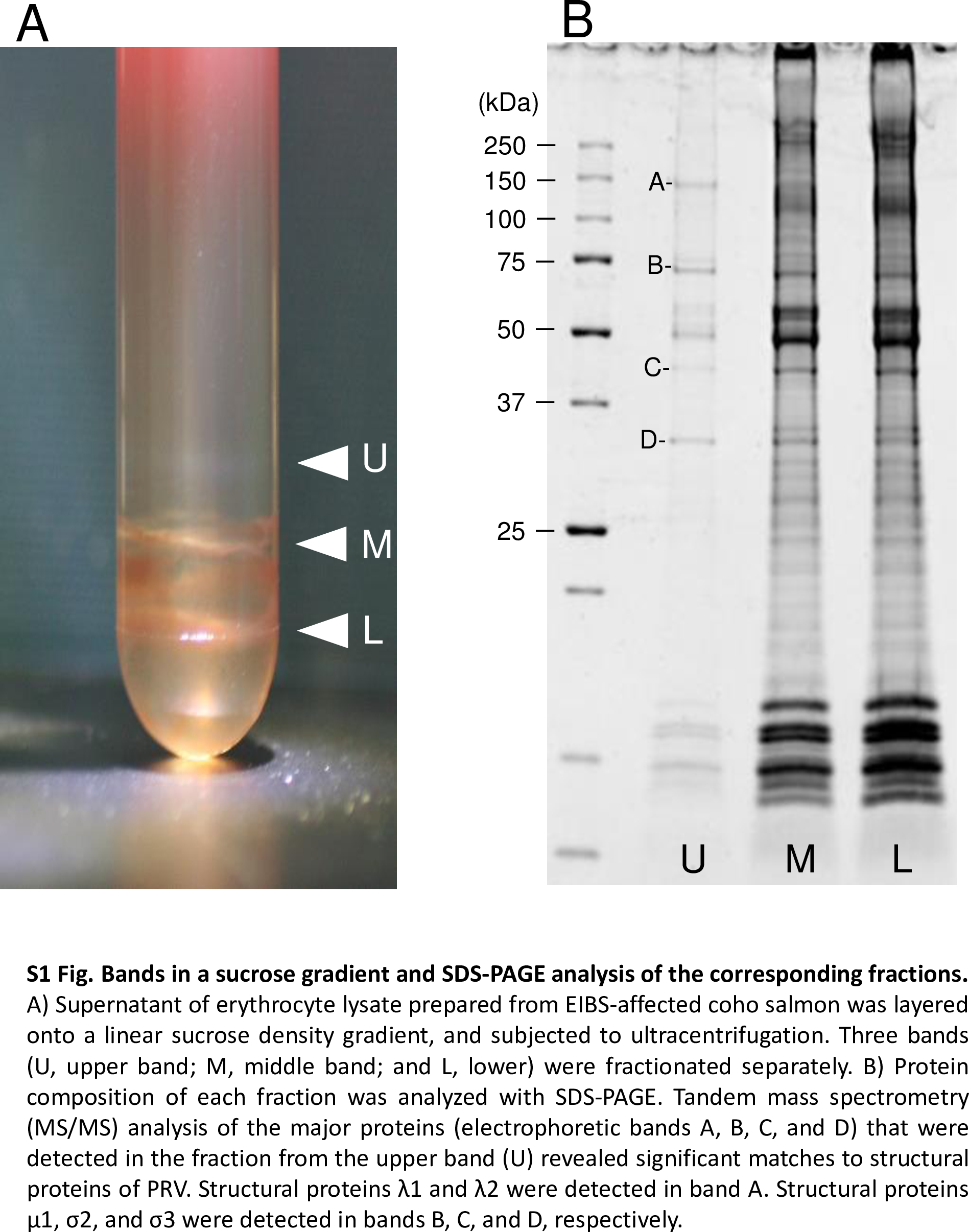

Supplement: S1 Fig — A) Supernatant of erythrocyte lysate prepared from EIBS-affected coho salmon was layered onto a linear sucrose density gradient, and subjected to ultracentrifugation. Three bands (U, upper band; M, middle band; and L, lower) were fractionated separately. B) Protein composition of each fraction was analyzed with SDS-PAGE. Tandem mass spectrometry (MS/MS) analysis of the major proteins (electrophoretic bands A, B, C, and D) that were detected in the fraction from the upper band (U) revealed significant matches to structural proteins of PRV. Structural proteins λ1 and λ2 were detected in band A. Structural proteins μ1, σ2, and σ3 were detected in bands B, C, and D, respectively. (TIF) [file pone.0165424.s001.tif]

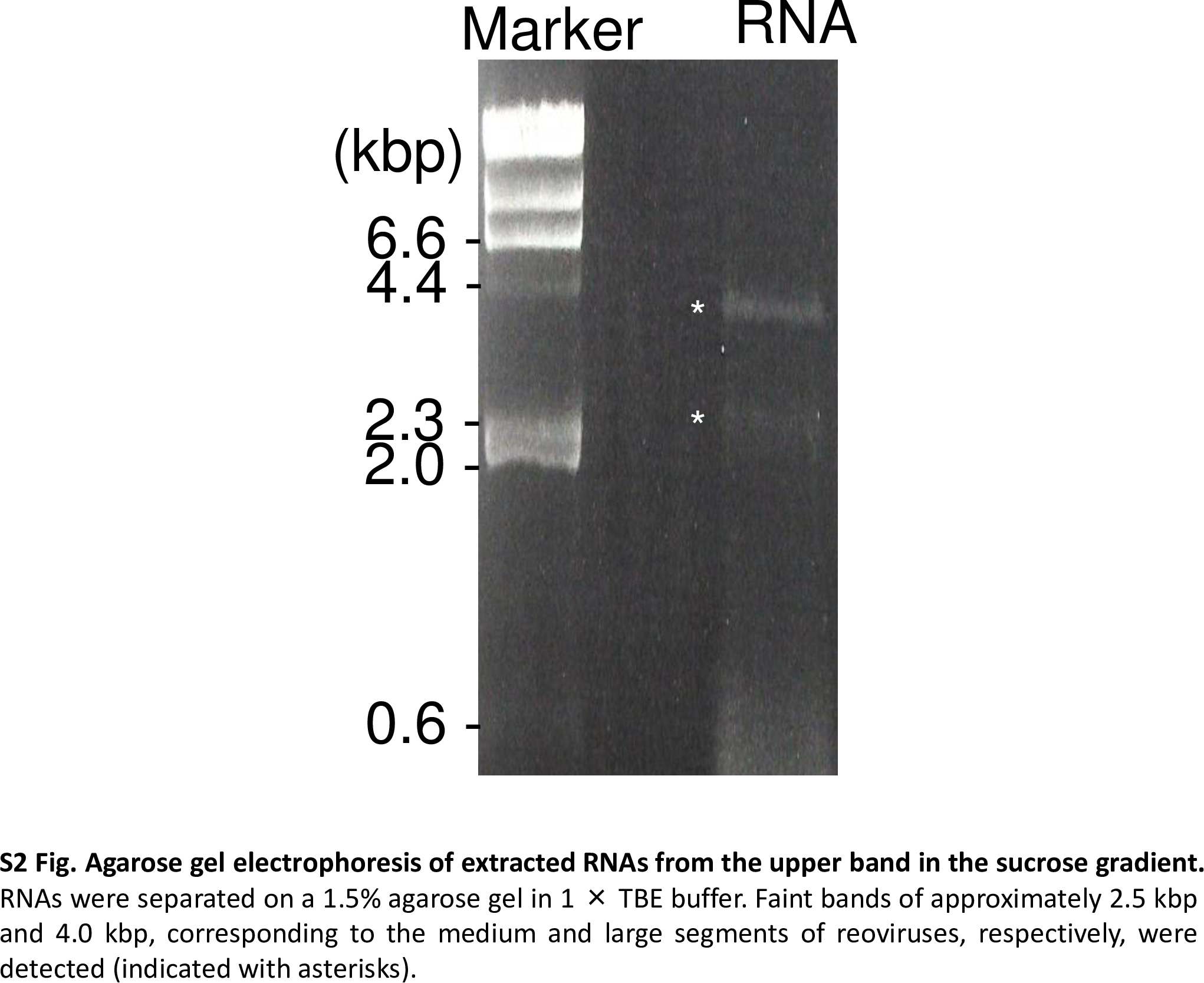

Supplement: S2 Fig — RNAs were separated on a 1.5% agarose gel in 1 × TBE buffer. Faint bands of approximately 2.5 kbp and 4.0 kbp, corresponding to the medium and large segments of reoviruses, respectively, were detected (indicated with asterisks). (TIF) [file pone.0165424.s002.tif]

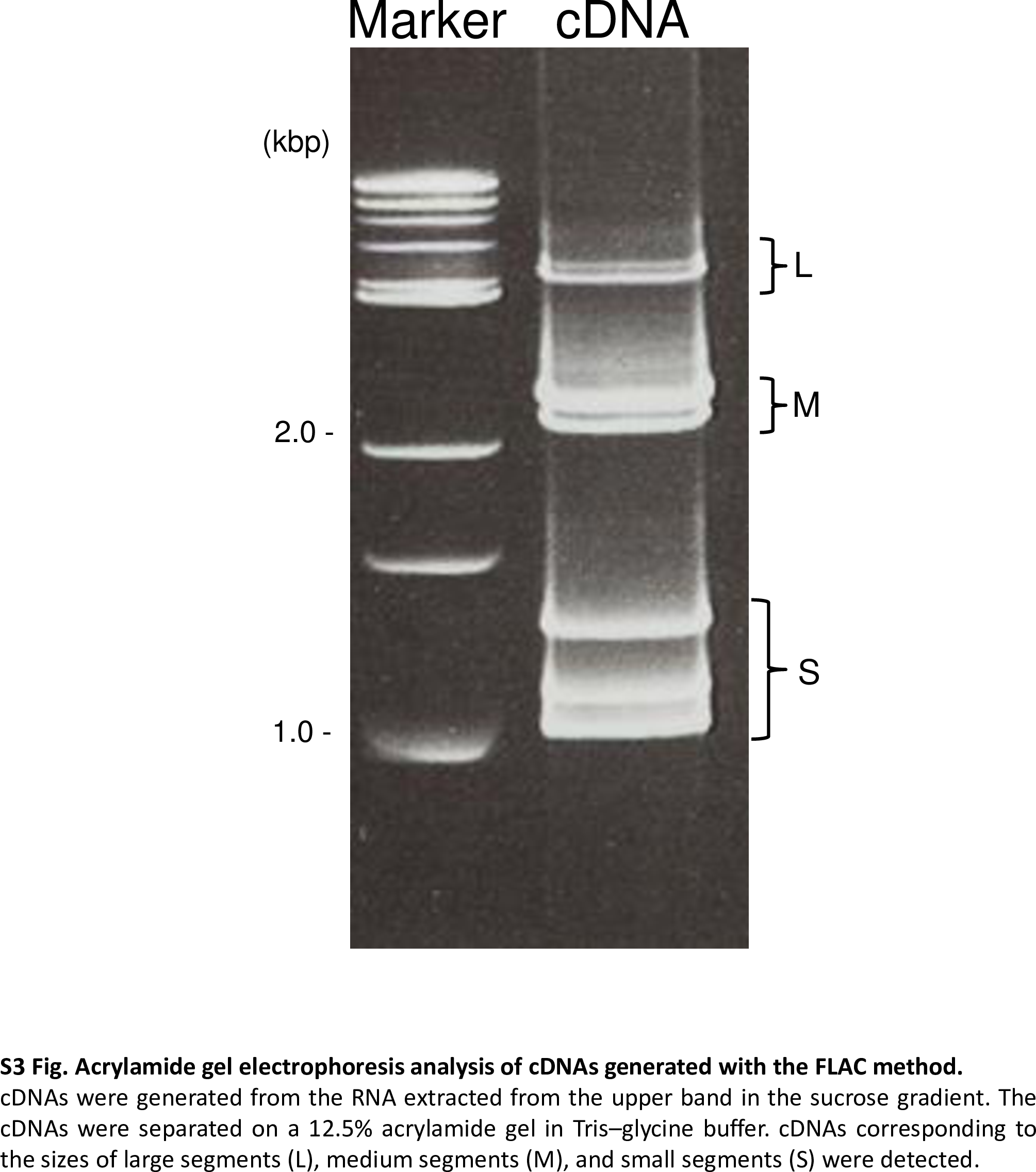

Supplement: S3 Fig — cDNAs were generated from the RNA extracted from the upper band in the sucrose gradient. The cDNAs were separated on a 12.5% acrylamide gel in Tris–glycine buffer. cDNAs corresponding to the sizes of large segments (L), medium segments (M), and small segments (S) were detected. (TIF) [file pone.0165424.s003.tif]

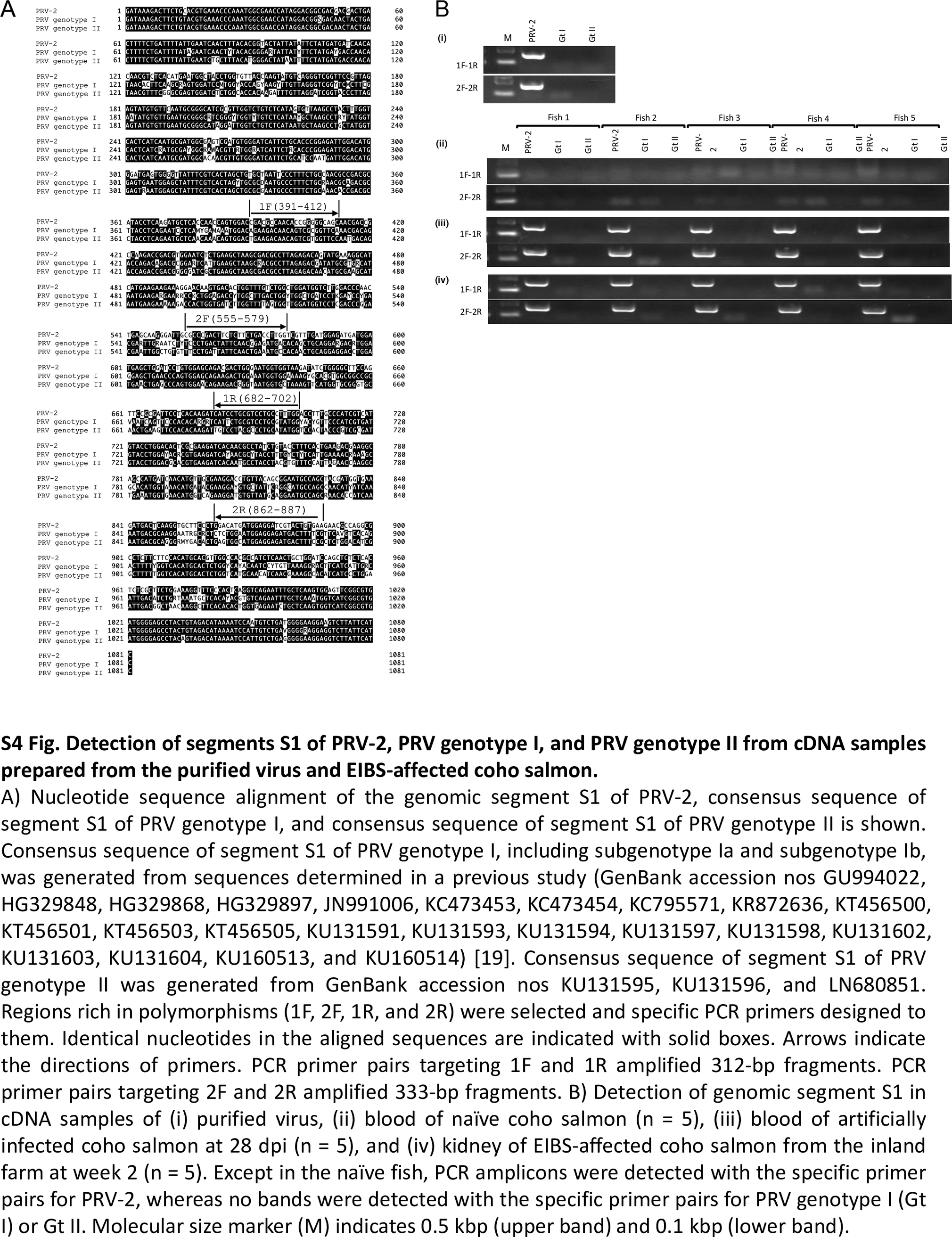

Supplement: S4 Fig — A) Nucleotide sequence alignment of the genomic segment S1 of PRV-2, consensus sequence of segment S1 of PRV genotype I, and consensus sequence of segment S1 of PRV genotype II is shown. Consensus sequence of segment S1 of PRV genotype I, including subgenotype Ia and subgenotype Ib, was generated from sequences determined in a previous study (GenBank accession nos GU994022, HG329848, HG329868, HG329897, JN991006, KC473453, KC473454, KC795571, KR872636, KT456500, KT456501, KT456503, KT456505, KU131591, KU131593, KU131594, KU131597, KU131598, KU131602, KU131603, KU131604, KU160513, and KU160514) [19]. Consensus sequence of segment S1 of PRV genotype II was generated from GenBank accession nos KU131595, KU131596, and LN680851. Regions rich in polymorphisms (1F, 2F, 1R, and 2R) were selected and specific PCR primers designed to them. Identical nucleotides in the aligned sequences are indicated with solid boxes. Arrows indicate the directions of primers. PCR primer pairs targeting 1F and 1R amplified 312-bp fragments. PCR primer pairs targeting 2F and 2R amplified 333-bp fragments. B) Detection of genomic segment S1 in cDNA samples of (i) purified virus, (ii) blood of naïve coho salmon (n = 5), (iii) blood of artificially infected coho salmon at 28 dpi (n = 5), and (iv) kidney of EIBS-affected coho salmon from the inland farm at week 2 (n = 5). Except in the naïve fish, PCR amplicons were detected with the specific primer pairs for PRV-2, whereas no bands were detected with the specific primer pairs for PRV genotype I (Gt I) or Gt II. Molecular size marker (M) indicates 0.5 kbp (upper band) and 0.1 kbp (lower band). (TIF) [file pone.0165424.s004.tif]
