# Supplementary material for: Full-Genome Sequencing and Confirmation of the Causative Agent of Erythrocytic Inclusion Body Syndrome in Coho Salmon Identifies a New Type of Piscine Orthoreovirus
Source: PLoS One. 2016 Oct 27;11(10):e0165424. doi: 10.1371/journal.pone.0165424 (PMC5082797; doi:10.1371/journal.pone.0165424)
Supplement: S1 Table — (DOCX) [file pone.0165424.s005.docx]

| **S1 Table. List of accession numbers of proteins from representative viruses in the subfamily *Spinareovirinae* used in this study.** | | | | | | | | | |
| --- | --- | --- | --- | --- | --- | --- | --- | --- | --- |
| Protein*^1^ | | PRV-2 | PRV (genotype Ia) | PRV (genotype Ib) | LMBRV | GCRV104 | ARV | MRV | MAHLV |
| λ1 | (Core shell) | LC145610 | AGR27924 | AGR44268 | ANY92090 | AFG73674 | ACH72474 | AAD42304 | YP_009246471 |
| λ2 | (Core turret) | LC145608 | AGR27925 | AGR44266 | ANY92091 | AFG73672 | ACH72478 | AAK57507 | YP_009246465 |
| λ3 | (Core RdRp) | LC145609 | AGR27926 | AGR44279 | ANY92092 | AFG73673 | ACH72476 | AAA47234 | YP_009246466 |
| μ2 | (Core NTPase) | LC145612 | AGR27927 | AGR44278 | ANY92093 | AFG73676 | AAT52025 | AAL99936 | YP_009246467 |
| μ1 | (Outer shell) | LC145613 | AGR27928 | AGR44280 | ANY92094 | AFG73677 | AAW78486 | AAM10735 | YP_009246472 |
| μNS | (NS factory) | LC145611 | AGR27929 | AGR44281 | ANY92095 | AFG73675 | AAT52027 | AAF13169 | YP_009246468 |
| σ3 | (Outer clamp) | LC145616 | AGR27897 | AGR44282 | ANY92098 | AFG73680 | AAC18125 | CAA43783 | YP_009246473 |
| σ1s | (NS, p13) | LC145616 | AGR27896 | AGR44283 | ANY92097 | - | - | - | - |
| σ2 | (Core clamp) | LC145614 | AGR27930 | AGR44284 | ANY92096 | ADM25848 | AAC18121 | AAA47239 | YP_009246469 |
| σNS | (NS RNA) | LC145615 | AGR27931 | AGR44285 | ANY92099 | AFG73679 | AAC18129 | AAA47281 | YP_009246470 |
| σ1 | (Outer fiber) | LC145617 | AGR27932 | AGR44286 | ANY92100 | AFG73678 | AAF45153 | AAA47242 | - |
|  |  |  |  |  |  |  |  |  |  |
| *^1^ Protein names assigned in the previous reports [26, 27] are shown in parentheses. | | | | | | | | |  |
